# Current Understanding of RAD52 Functions: Fundamental and Therapeutic Insights

**DOI:** 10.3390/cancers12030705

**Published:** 2020-03-17

**Authors:** Vanesa Gottifredi, Lisa Wiesmüller

**Affiliations:** 1 Fundación Instituto Leloir, IIBBA-Consejo Nacional de Investigaciones Científicas y Técnicas. Av. Patricias Argentinas 435, 1405 Buenos Aires, Argentina; 2 Division of Gynecological Oncology, Department of Obstetrics and Gynecology of the University of Ulm Prittwitzstrasse 43, 89075 Ulm, Germany

**Keywords:** DNA double-strand break repair, common fragile site, stalled replication fork, telomeres, fork reversal, R loops, nucleases, genome integrity

## Abstract

In this Special Issue, we would like to focus on the various functions of the RAD52 helicase-like protein and the current implications of such findings for cancer treatment. Over the last few years, various laboratories have discovered particular activities of mammalian RAD52—both in S and M phase—that are distinct from the auxiliary role of yeast RAD52 in homologous recombination. At DNA double-strand breaks, RAD52 was demonstrated to spur alternative pathways to compensate for the loss of homologous recombination functions. At collapsed replication forks, RAD52 activates break-induced replication. In the M phase, RAD52 promotes the finalization of DNA replication. Its compensatory role in the resolution of DNA double-strand breaks has put RAD52 in the focus of synthetic lethal strategies, which is particularly relevant for cancer treatment.

## 1. Introduction

This Special Issue gathers experts in the field to convey both comprehensive, insightful, and current perspectives on the different functions of human RAD52 in the maintenance of genomic integrity and the current implications of such findings for cancer treatment. Historically, RAD52 was described as an auxiliary factor of RAD51 in homologous recombination (HR) in yeast [1]. Around 2000, it became clear that in mammalian cells, BRCA2 had taken over the RAD51-chaperoning activity, stimulating filament formation on single-stranded DNA (ssDNA), while RAD52 was left with the single-strand annealing (SSA) activity [2]. Later, limited contributions of RAD52 to the repair of DNA double-strand breaks (DSBs)—such as during alternative non-homologous end-joining (A-NHEJ) and in compensating for the loss of HR functions—were also detected [3].

Full-length RAD52 forms a heptameric ring, whereby N- and C-terminal parts of each monomer form a positively charged ssDNA binding groove around the heptamer [4,5]. Oligomerization in the cytoplasm stimulates nuclear import, granted by the combined action of individually weak nuclear localization signals. Its RPA binding domains underlie RAD52´s biochemical functions in binding RPA-coated ssDNA, annealing, and homology-directed repair. Even though many details regarding the contribution of posttranslational modification to the function of RAD52 remain to be explored, we know that RAD52 acetylation is required for its accumulation at DSBs, sumoylation of yeast RAD52 for the choice of SSA over canonical recombination at repeats, and tyrosine phosphorylation of RAD52 for ssDNA, rather than dsDNA binding and the choice of SSA [6,7,8,9]. 

Over the last years, new functions of RAD52 have been identified. For example, various laboratories have discovered the involvement of mammalian RAD52 and RNA templates and RNA-DNA hybrid structures like R loops in unprecedented homology-directed DSB repair events [9]. First, RNA was discovered to serve as a bridging template in RAD52- and RPA-mediated homology-directed DSB repair, which may play a role during transcription, replication, class-switch recombination, and at telomeres. Second, in transcription-coupled HR (TC-HR) in G0/G1 cells, R loops generated during transcription were found to be bound by CSB, followed by RAD52-mediated, BRCA-independent RAD51 recruitment, and HR. Third, in transcription-activated HR (TA-HR) in S/G2 cells, RAD52 recruits the XPG cleaving R loops, which generates ssDNA overhangs for BRCA-dependent HR [9]. 

**Figure 1:**
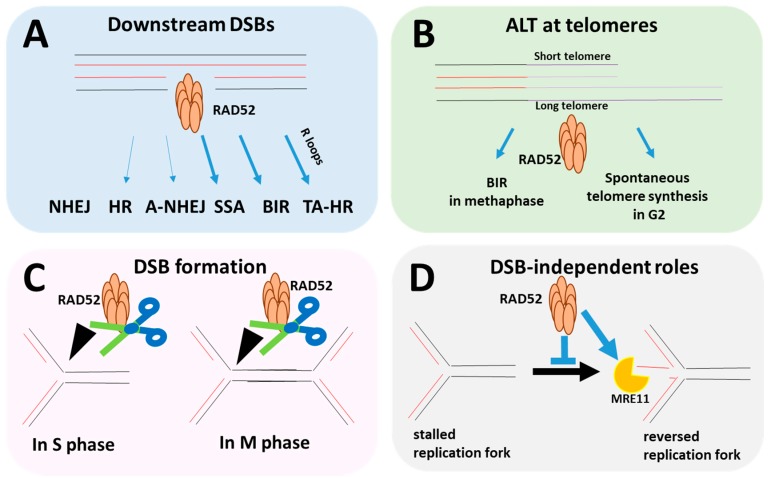
Multiple roles of RAD52 during DNA replication and repair. (**A**) RAD52 participates in various DNA double-strand break (DSB) repair processes by means of its strand-annealing activities. In some pathways, RAD52 acts as a backup factor (e.g., HR), while in others, it is absolutely required, e.g. single-strand annealing (SSA) at DSBs and break-induced replication (BIR) at single-ended DSBs (which are not shown in this scheme) [8,9,13], (**B**) RAD52 participates in the alternative lengthening of telomeres. RAD52 plays a role in BIR-mediated elongation of telomeres during pro-metaphase, but also promotes spontaneous telomere elongation in G2, independently of the SLX4 nuclease [11,14,15]. (**C**) RAD52 has also been implicated in the facilitation of DSB formation by MUS81 in Chk1-depleted cells and during MiDAS [10,16,17]. (**D**) DSB-independent roles of RAD52 were also reported in S phase. RAD52 prevents unleashed fork reversal, but once forks have reversed, it can facilitate MRE11-dependent degradation of newly synthesized DNA [12,18,19]. Template DNA: black, copied strand: red and violet strands: telomeric regions.

Surprisingly, RAD52 also acts during events other than canonical DSB repair—namely, during DNA replication when it counteracts excessive fork regression, which may exhaust protection factors, causing breakage of reversed forks. At collapsed replication forks, RAD52 activates break-induced replication (BIR), a specialized pathway that repairs single-ended DSBs. During M phase, RAD52-mediated BIR also promotes the finalization of DNA replication (MiDAS- mitotic DNA synthesis). Notably, RAD52 cooperates with various nucleases, namely, MUS81 during BIR, ERCC1/XPF during SSA, and XPG during TA-HR. It also cooperates with MRE11 and MUS81/EME1 at de-protected forks that have reverted to process these structures into HR substrates, ultimately enabling the cell to continue DNA replication [10,11,12]. The multiple activities of RAD52 known to date are summarized in Figure 1.

Therefore, compensatory roles in the resolution of DSBs, as well as at replication forks, put RAD52 in the focus of synthetic lethal strategies, which is particularly relevant for cancer treatment. Consequently, efforts were made to identify RAD52-inhibitory compounds to kill HR-defective tumor cells in a synthetic lethal fashion [8]. The specific mode of action of these compounds is determined by the above-mentioned biochemical features of RAD52 so that, e.g., interfering with heptamer formation blocks recruitment of RAD52 to DNA damage sites, while the occupation of the ssDNA binding groove abrogates ssDNA binding. In the light of specific effects of drugs targeting other DNA repair proteins, such as PARP1-trapping compounds [20], it will be interesting to see whether inactivation of specific activities of RAD52 will be more toxic than complete loss-of-function of RAD52.

Below, readers can find a sneak peek of the subjects covered by each review.

The review entitled, “When RAD52 entitles mitosis to accept unscheduled DNA synthesis” by Camille Franchet and Jean Sébastien Hoffmann discusses the discovery of MiDAS from a historical perspective [11]. DNA synthesis in the G2/M phase was first reported in cells depleted of the specialized DNA polymerase, pol η [21]. Because pol η facilitates the synthesis of DNA at hard-to-replicate regions such as common fragile sites and telomeres, its loss revealed unscheduled DNA synthesis outside the S phase. Later on, the Hickson laboratory demonstrated that MiDAS can be easily revealed upon mild DNA replication stress [22]. Such DNA duplication is achieved by BIR and depends on RAD52 [23]. MiDAS can take place in M phase and at late stages of the next S phase if under-replicated DNA is shielded by 53BP1 during G1 phase [24]. Conceptually, such findings were paradigm-breaking because the traditional notion of the S phase ending before the start of the G2 phase was demolished. The review also discusses the scenarios in which MIDAS is needed, the molecular effectors of such pathways, and the consequences of its loss. Remarkably, the review compares MiDAS events at CFS and at telomeric regions, pointing out that while an endonuclease cut must precede the former, it is unclear if such DNA processing is absolutely required for at least some variants of alternative lengthening of telomeres (ALT). In particular, it should be pointed out that a non-epistatic effect between RAD52 and the nucleases scaffold SLX4 during ALT has been reported [14], suggesting that RAD52 may act on telomeres in a manner that is independent of nucleases associated with SLX4. The authors also discuss the potential addiction of cancer cells to MiDAS and the potential improvement that MiDAS inhibition could provide to current therapies, particularly considering the limited role that MiDAS may have in the protection of the genome of normal cells [25]. 

The review by Xiaohua Wu entitled, “Replication stress response links RAD52 to protecting common fragile sites (CFS)” puts the spotlight on chromosomal regions [10] that are prone to break upon mild replication stress [26]. Because of their fragility, it has been proposed that CFS instability is a driving force for tumorigenesis [27,28,29]. Such a propensity to break may result from a combination of many replication challenges accumulating at CFS, including late replication timing, few replication origins, excess replication stalling by AT-rich sequences, and collision between replication and transcription [10]. Because of such challenges, CFS can frequently break in S phase. Xiaohua Wu discusses how the secondary DNA structures of DSBs generated at CFS trigger HR events that depend on RAD52 [30]. Such a strong dependency on RAD52 may result from a role of RAD52 in the facilitation of nuclease-mediated removal of the secondary structure, the promotion of second end capture, and/or the initiation of BIR [31,32,33,34,35]. Repair of such a subtype of DSBs is expected to occur in the S phase [30,34]. On the other hand, RAD52 may also promote DSB formation at CFS when cells enter M phase with under-replicated DNA. In such scenarios, to minimize mitotic missegregation, RAD52 facilitates nuclease-mediated cleavage of under-replicated DNA in mitotic prophase, promoting the formation of a single-ended DSB, a DNA structure that can be processed by BIR [16,36]. Such subtypes of M-phase BIR or MiDAS require the annealing activity of RAD52 to initiate this unidirectional DNA synthesis [22]. Xiaohua Wu also discusses reasons why to consider RAD52 as a potential target for cancer therapies, which genetics backgrounds might benefit from this treatment, and which limitations exist in the design of appropriate RAD52 inhibitors [28,37].

The review by Maria Spies, Pietro Pichierri, and colleagues entitled, “Physiological and Pathological Roles of RAD52 at DNA Replication” contains entire sections devoted to discussing functions of RAD52 that are independent from the resolution of DSBs, but may influence their formation [12]. One such function is a gatekeeper role of RAD52 at DNA replication forks, which prevents their excessive reversal by SMARCAL1 (SWI/SNF Related, Matrix Associated, Actin Dependent Regulator of Chromatin, Subfamily A-Like 1). In the absence of RAD52, unleashed fork reversal events lead to the exhaustion of RAD51, which in turn triggers unscheduled degradation by MRE11 [19]. Conversely, in forks that have already reversed, RAD52 facilitates MRE11-mediated nascent DNA degradation [18]. These observations suggest that once loaded at stall forks, RAD52 may perform different and apparently opposed functions, before and upon fork reversal. Since many other proteins also regulate fork reversal [12], the authors thoroughly compared previously described modes of action with the one of RAD52. Also, while the synthetic lethality reported for BRCA2 and RAD52 has been ascribed to the contribution of RAD52 to the repair of DSB [38,39], this review speculates on the contribution that DSB-independent functions of RAD52 may have on such synthetic lethality. Another DSB repair-independent function of RAD52 discussed in depth is its ability to stimulate DSB formation by promoting the loading of MUS81 [17,37,40,41]. The potential identity of the substrates of the RAD52/MUS81 complex is examined as well. The final section is devoted to discussing potential clinical implications of RAD52 inhibition. As RAD52 is overexpressed in tumors, the authors underscore the potential effect that more profound knowledge of RAD52 functions may have when predicting the benefit of RAD52 inhibition for cancer patients undergoing single and combined treatment regimens.

In the review entitled, “Emerging roles of RAD52 in genome maintenance” Manisha Jalam, Kyrie Olsen and Simon Powell concentrate on the functions of RAD52 that are relevant to mammals [9]. After BRCA2 took center stage from yeast Rad52 in our understanding of aiding RAD51 filament formation during homologous recombination (HR) [2]. mammalian RAD52 remained underexplored for years. Simon Powell and his team awakened this sleeping beauty upon realizing the great therapeutic potential of synthetic lethality after RAD52 loss-of-function in HR-deficient cells [38]. Thereupon, RAD52 became a target for therapy in BRCA-deficient tumors, spurring screening efforts for identification of pharmacological RAD52 inhibitors. In parallel, a plethora of RAD52 functions in mammalian cells beyond its canonical DSB repair activity in the SSA pathway were recognized. In their review, the authors provide a comprehensive overview of RAD52´s non-canonical roles in DNA repair during the cell cycle, i.e., backup functions in HR, BIR, ALT, and processing of stalled forks in S/G2 phase, MiDAS in M phase, and TC-HR in G1 [9]. The authors then focus on more recent discoveries of non-canonical RAD52 functions in RNA-templated repair and R-loop processing, which again were prompted by observations made in yeast [42,43]. When the authors sorted the relative affinities of human RAD52 for different nucleic acid structures, it became evident why RNA can replace ssDNA efficiently as a template during HR, and why RAD52 interacts with RNA-DNA hybrids. To fully understand the biology of RAD52 as a therapeutic target, attention must be focused on RAD52´s prominent yet understudied roles in RNA-DNA hybrid structures, such as in transcription-coupled repair, engaging RAD52, rather than BRCA1/BRCA2 in loading RAD51. The authors propose that, in fact, concomitant disruption of these multiple activities on DNA and RNA is necessary for the synthetic lethal effect of RAD52 inactivation in tumor cells displaying BRCAness. 

In the review, “RAD52: viral friend or foe?” Eric Hendrickson evaluates the contribution of RAD52 to viral replication events [13]. Eric Hendrickson made an important contribution to the field when he applied reporter-based DSB repair assays on human RAD52 knockout cells, demonstrating that RAD52 also acts in homology-directed DSB repair pathways other than SSA, as well as that additional RAD52-independent SSA mechanisms must exist in human cells [3]. The same tools also revealed that adeno-associated virus (AAV) depends on RAD52 for random genome integration. In light of the small size of viral genomes, it is clear why hijacking host proteins for DNA recombination and replication is a universal strategy of viruses to integrate, amplify, or protect their genomes from cellular defense mechanisms [44]. In his review, Eric Hendrickson provides an overview of the most diverse roles of RAD52 in the life cycle of different viruses [13]. Thus, the ssDNA virus AAV seems to utilize the host factor RAD52 to reanneal the only necessary cis-acting sequence—the inverted terminal repeat (ITR) secondary structures after unidirectional replication of the viral DNA— priming thereafter the next round of viral replication. RAD52 binds to these ITRs, and is hypothesized to promote AAV concatemerization by SSA and viral integration into the cellular genomes by A-NHEJ, an SSA-related process engaging microhomologies. RAD52 also binds retroviral long terminal repeats (LTRs) and promotes the annealing step between LTRs for unidirectional replication of human immunodeficiency virus (HIV) [45]. Counterintuitively, RAD52 was shown to exert antiviral effects, which may be explained by the production of non-productive viral cDNA circles. In contrast with the situation with AAV, RAD52 does not promote HIV integration [46], which may explain the essential role of RAD52 in the AAV, versus the HIV life cycle. The varying roles of RAD52 in the replication of different viruses provide further hints for the functional adaptation of RAD52 to the cellular requirements, such as also highlighted by the pathway-specialization in HR in yeast, versus SSA in mammals. 

In their review entitled, “RAD52 as a potential target of Synthetic lethal-based anticancer therapies” Tomasz Skorski and his team discuss the impact of RAD52-dependent alternative repair pathways as a basis of personalized cancer therapies [8]. HR-deficient tumor cells accumulate DSBs at collapsed replication forks and shift to alternative DSB repair pathways, RAD52-dependent SSA in particular; BRCA2-defects in loading RAD51 can partially be backed by RAD52 [47]. Another advantage of this strategy will be that HR represents the DSB repair pathway with the highest fidelity, whereas SSA is always mutagenic, deleting intervening sequences between the annealed repeats. Moreover, RAD52 stabilizes and restores stalled or collapsed replication forks in alternative pathways, as compared with RAD51, BRCA1, and BRCA2 (see above [19]). Therefore, inactivation of both RAD52 functions in HR-deficient cells is a prime example for the definition of synthetic lethality, as survival depends on the alternative pathways at both DSBs and forks. Limitations may arise from the fact that even further alternative pathways exist, such as A-NHEJ mechanisms depending on PARP1 [48,49], why simultaneous inhibition of RAD52 and PARP1 in a so-called dual synthetic lethality approach has emerged as a new strategy. The authors discuss the chemical classes of so far reported inhibitors of human RAD52 with demonstrated efficiency as tools to exploit the principle of synthetic lethality in cells with BRCAness: While F79 interferes with DNA binding by RAD52 in general, A5MP, AICAR, and F779-0434 inhibit ssDNA-binding, D-103 RAD52-mediated D-loop formation, and SSA. Epigallocatechin compounds and corilagin inhibit wrapping of ssDNA around the RAD52 oligomer. 6-OH-dopa is an interesting compound for experimental approaches, as it disrupts the formation of the RAD52 heptamer, affecting SSA quite specifically. Aside from breast cancer and pancreatic adenocarcinoma cells displaying BRCAness, the compounds were tested in leukemia cells with oncogenic BCR-ABL1 kinase, promoting SSA [50]. 

## 2. Conclusions

RAD52 research has seen a renaissance in recent years after the disappointment linked to the discovery of mammalian RAD52 playing a subordinate role to BRCA2 in human cells, i.e., an at-most minor role in HR. RAD52 re-entered the stage when it became clear that the principle of synthetic lethality may be applied to HR-deficient tumors treated with RAD52 inhibitory molecules. Given that several viruses also rely on RAD52 during their life cycle, RAD52 inhibitors also represent an Achilles heel for these, and as-yet-undescribed viruses. Therefore, RAD52 inhibitors may be promising candidates for research on antiviral drug development as well. Moreover, recent research has uncovered an unanticipated diversity of RAD52 functions on RNA, DNA, RNA-DNA hybrids, R loops, replication forks, under-replicated DNA, telomeres, and DSBs that encompass both genome-destabilizing and stabilizing activities. Therefore, the definition of pharmacological applications of RAD52 inhibitors will require careful evaluations of various endpoints before clinical trials can reasonably be initiated. 

## References

[1] Sugiyama T., New J.H., Kowalczykowski S.C. (1998). DNA annealing by RAD52 protein is stimulated by specific interaction with the complex of replication protein A and single-stranded DNA. Proc. Natl. Acad. Sci. USA.

[2] Stark J.M., Pierce A.J., Oh J., Pastink A., Jasin M. (2004). Genetic steps of mammalian homologous repair with distinct mutagenic consequences. Mol. Cell Biol..

[3] Kan Y., Batada N.N., Hendrickson E.A. (2017). Human somatic cells deficient for RAD52 are impaired for viral integration and compromised for most aspects of homology-directed repair. DNA Repair (Amst).

[4] Stasiak A.Z., Larquet E., Stasiak A., Muller S., Engel A., Van Dyck E., West S.C., Egelman E.H. (2000). The human Rad52 protein exists as a heptameric ring. Curr. Biol..

[5] Kagawa W., Kagawa A., Saito K., Ikawa S., Shibata T., Kurumizaka H., Yokoyama S. (2008). Identification of a second DNA binding site in the human Rad52 protein. J. Biol. Chem..

[6] Altmannova V., Eckert-Boulet N., Arneric M., Kolesar P., Chaloupkova R., Damborsky J., Sung P., Zhao X., Lisby M., Krejci L. (2010). Rad52 SUMOylation affects the efficiency of the DNA repair. Nucleic Acids Res..

[7] Yasuda T., Kagawa W., Ogi T., Kato T.A., Suzuki T., Dohmae N., Takizawa K., Nakazawa Y., Genet M.D., Saotome M. (2018). Novel function of HATs and HDACs in homologous recombination through acetylation of human RAD52 at double-strand break sites. PLoS Genet..

[8] Toma M., Sullivan-Reed K., Sliwinski T., Skorski T. (2019). RAD52 as a Potential Target for Synthetic Lethality-Based Anticancer Therapies. Cancers (Basel).

[9] Jalan M., Olsen K.S., Powell S.N. (2019). Emerging Roles of RAD52 in Genome Maintenance. Cancers (Basel).

[10] Wu X. (2019). Replication Stress Response Links RAD52 to Protecting Common Fragile Sites. Cancers (Basel).

[11] Franchet C., Hoffmann J.S. (2019). When RAD52 Allows Mitosis to Accept Unscheduled DNA Synthesis. Cancers (Basel).

[12] Malacaria E., Honda M., Franchitto A., Spies M., Pichierri P. (2020). Physiological and Pathological Roles of RAD52 at DNA Replication Forks. Cancers (Basel).

[13] Hendrickson E.A. (2020). RAD52: Viral Friend or Foe?. Cancers (Basel).

[14] Verma P., Dilley R.L., Zhang T., Gyparaki M.T., Li Y., Greenberg R.A. (2019). RAD52 and SLX4 act nonepistatically to ensure telomere stability during alternative telomere lengthening. Genes Dev..

[15] Zhang J.M., Yadav T., Ouyang J., Lan L., Zou L. (2019). Alternative Lengthening of Telomeres through Two Distinct Break-Induced Replication Pathways. Cell Rep..

[16] Bhowmick R., Minocherhomji S., Hickson I.D. (2016). RAD52 Facilitates Mitotic DNA Synthesis Following Replication Stress. Mol. Cell.

[17] Murfuni I., Basile G., Subramanyam S., Malacaria E., Bignami M., Spies M., Franchitto A., Pichierri P. (2013). Survival of the replication checkpoint deficient cells requires MUS81-RAD52 function. PLoS Genet..

[18] Mijic S., Zellweger R., Chappidi N., Berti M., Jacobs K., Mutreja K., Ursich S., Ray Chaudhuri A., Nussenzweig A., Janscak P. (2017). Replication fork reversal triggers fork degradation in BRCA2-defective cells. Nat. Commun..

[19] Murfuni I., Basile G., Subramanyam S., Malacaria E., Bignami M., Spies M., Franchitto A., Pichierri P. (2019). Author Correction: Rad52 prevents excessive replication fork reversal and protects from nascent strand degradation. Nat. Commun..

[20] Isakoff S.J., Puhalla S., Domchek S.M., Friedlander M., Kaufman B., Robson M., Telli M.L., Dieras V., Han H.S., Garber J.E. (2017). A randomized Phase II study of veliparib with temozolomide or carboplatin/paclitaxel versus placebo with carboplatin/paclitaxel in BRCA1/2 metastatic breast cancer: design and rationale. Future Oncol..

[21] Bergoglio V., Boyer A.S., Walsh E., Naim V., Legube G., Lee M.Y., Rey L., Rosselli F., Cazaux C., Eckert K.A. (2013). DNA synthesis by Pol eta promotes fragile site stability by preventing under-replicated DNA in mitosis. J. Cell Biol..

[22] Minocherhomji S., Ying S., Bjerregaard V.A., Bursomanno S., Aleliunaite A., Wu W., Mankouri H.W., Shen H., Liu Y., Hickson I.D. (2015). Replication stress activates DNA repair synthesis in mitosis. Nature.

[23] Ozer O., Bhowmick R., Liu Y., Hickson I.D. (2018). Human cancer cells utilize mitotic DNA synthesis to resist replication stress at telomeres regardless of their telomere maintenance mechanism. Oncotarget.

[24] Lukas C., Savic V., Bekker-Jensen S., Doil C., Neumann B., Pedersen R.S., Grofte M., Chan K.L., Hickson I.D., Bartek J. (2011). 53BP1 nuclear bodies form around DNA lesions generated by mitotic transmission of chromosomes under replication stress. Nat. Cell Biol..

[25] Lok B.H., Powell S.N. (2012). Molecular pathways: understanding the role of Rad52 in homologous recombination for therapeutic advancement. Clin. Cancer Res..

[26] Durkin S.G., Glover T.W. (2007). Chromosome fragile sites. Annu. Rev. Genet..

[27] Gorgoulis V.G., Vassiliou L.V., Karakaidos P., Zacharatos P., Kotsinas A., Liloglou T., Venere M., Ditullio R.A., Kastrinakis N.G., Levy B. (2005). Activation of the DNA damage checkpoint and genomic instability in human precancerous lesions. Nature.

[28] Tsantoulis P.K., Kotsinas A., Sfikakis P.P., Evangelou K., Sideridou M., Levy B., Mo L., Kittas C., Wu X.R., Papavassiliou A.G. (2008). Oncogene-induced replication stress preferentially targets common fragile sites in preneoplastic lesions. A genome-wide study. Oncogene.

[29] Bartkova J., Horejsi Z., Koed K., Kramer A., Tort F., Zieger K., Guldberg P., Sehested M., Nesland J.M., Lukas C. (2005). DNA damage response as a candidate anti-cancer barrier in early human tumorigenesis. Nature.

[30] Wang H., Li S., Oaks J., Ren J., Li L., Wu X. (2018). The concerted roles of FANCM and Rad52 in the protection of common fragile sites. Nat. Commun..

[31] Mazina O.M., Keskin H., Hanamshet K., Storici F., Mazin A.V. (2017). Rad52 Inverse Strand Exchange Drives RNA-Templated DNA Double-Strand Break Repair. Mol. Cell.

[32] Ma C.J., Kwon Y., Sung P., Greene E.C. (2017). Human RAD52 interactions with replication protein A and the RAD51 presynaptic complex. J. Biol. Chem..

[33] Liu J., Heyer W.D. (2011). Who’s who in human recombination: BRCA2 and RAD52. Proc. Natl. Acad. Sci. USA.

[34] Li S., Lu H., Wang Z., Hu Q., Wang H., Xiang R., Chiba T., Wu X. (2019). ERCC1/XPF Is Important for Repair of DNA Double-Strand Breaks Containing Secondary Structures. iScience.

[35] Motycka T.A., Bessho T., Post S.M., Sung P., Tomkinson A.E. (2004). Physical and functional interaction between the XPF/ERCC1 endonuclease and hRad52. J. Biol. Chem..

[36] Naim V., Wilhelm T., Debatisse M., Rosselli F. (2013). ERCC1 and MUS81-EME1 promote sister chromatid separation by processing late replication intermediates at common fragile sites during mitosis. Nat. Cell Biol..

[37] Hengel S.R., Malacaria E., Folly da Silva Constantino L., Bain F.E., Diaz A., Koch B.G., Yu L., Wu M., Pichierri P., Spies M.A. (2016). Small-molecule inhibitors identify the RAD52-ssDNA interaction as critical for recovery from replication stress and for survival of BRCA2 deficient cells. Elife.

[38] Feng Z., Scott S.P., Bussen W., Sharma G.G., Guo G., Pandita T.K., Powell S.N. (2011). Rad52 inactivation is synthetically lethal with BRCA2 deficiency. Proc. Natl. Acad. Sci. USA.

[39] Lok B.H., Carley A.C., Tchang B., Powell S.N. (2013). RAD52 inactivation is synthetically lethal with deficiencies in BRCA1 and PALB2 in addition to BRCA2 through RAD51-mediated homologous recombination. Oncogene.

[40] Sotiriou S.K., Kamileri I., Lugli N., Evangelou K., Da-Re C., Huber F., Padayachy L., Tardy S., Nicati N.L., Barriot S. (2016). Mammalian RAD52 Functions in Break-Induced Replication Repair of Collapsed DNA Replication Forks. Mol. Cell.

[41] Doe C.L., Osman F., Dixon J., Whitby M.C. (2004). DNA repair by a Rad22-Mus81-dependent pathway that is independent of Rhp51. Nucleic Acids Res..

[42] Storici F., Bebenek K., Kunkel T.A., Gordenin D.A., Resnick M.A. (2007). RNA-templated DNA repair. Nature.

[43] Stirling P.C., Chan Y.A., Minaker S.W., Aristizabal M.J., Barrett I., Sipahimalani P., Kobor M.S., Hieter P. (2012). R-loop-mediated genome instability in mRNA cleavage and polyadenylation mutants. Genes Dev..

[44] Weller S.K., Sawitzke J.A. (2014). Recombination promoted by DNA viruses: phage lambda to herpes simplex virus. Annu. Rev. Microbiol..

[45] Lau A., Kanaar R., Jackson S.P., O’Connor M.J. (2004). Suppression of retroviral infection by the RAD52 DNA repair protein. EMBO J..

[46] Li L., Olvera J.M., Yoder K.E., Mitchell R.S., Butler S.L., Lieber M., Martin S.L., Bushman F.D. (2001). Role of the non-homologous DNA end joining pathway in the early steps of retroviral infection. EMBO J..

[47] Karanam K., Kafri R., Loewer A., Lahav G. (2012). Quantitative live cell imaging reveals a gradual shift between DNA repair mechanisms and a maximal use of HR in mid S phase. Mol. Cell.

[48] Audebert M., Salles B., Calsou P. (2004). Involvement of poly(ADP-ribose) polymerase-1 and XRCC1/DNA ligase III in an alternative route for DNA double-strand breaks rejoining. J. Biol. Chem..

[49] Wang M., Wu W., Wu W., Rosidi B., Zhang L., Wang H., Iliakis G. (2006). PARP-1 and Ku compete for repair of DNA double strand breaks by distinct NHEJ pathways. Nucleic Acids Res..

[50] Cramer K., Nieborowska-Skorska M., Koptyra M., Slupianek A., Penserga E.T., Eaves C.J., Aulitzky W., Skorski T. (2008). BCR/ABL and other kinases from chronic myeloproliferative disorders stimulate single-strand annealing, an unfaithful DNA double-strand break repair. Cancer Res..

